# Highly Sensitive Hall Sensors Based on Chemical Vapor Deposition Graphene

**DOI:** 10.1021/acsanm.3c03920

**Published:** 2023-11-21

**Authors:** Ayush Tyagi, Leonardo Martini, Zewdu M. Gebeyehu, Vaidotas Mišeikis, Camilla Coletti

**Affiliations:** † NEST, Scuola Normale Superiore, Piazza San Silvestro 12, 56127 Pisa, Italy; ‡ Center for Nanotechnology Innovation @NEST, Instituto Italiano di Technologia, Piazza San Silvestro 12, 56127 Pisa, Italy; § Graphene Laboratories, Istituto Italiano di Tecnologia, via Morego 30, 16163 Genova, Italy

**Keywords:** graphene, Hall sensors, scalability, sensitivity, stability

## Abstract

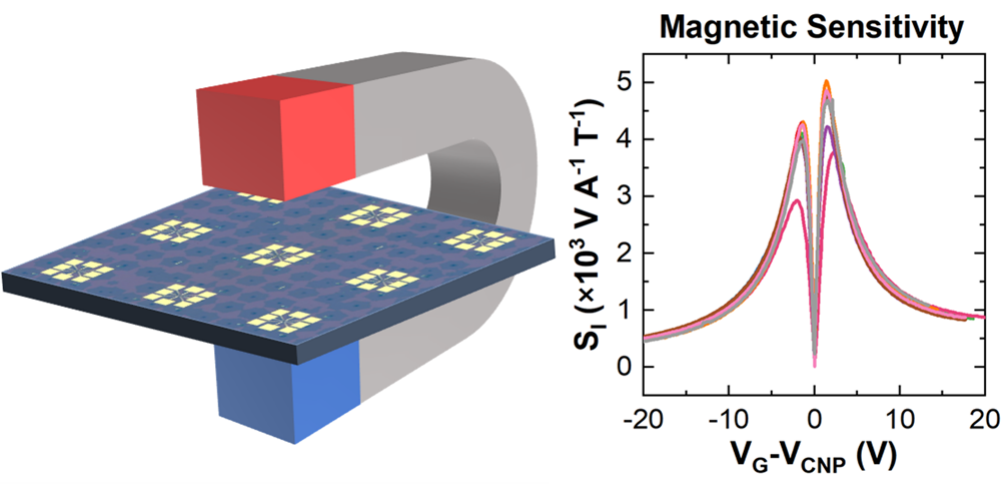

In this work, we demonstrate highly sensitive and scalable Hall sensors fabricated by adopting arrays of monolayer single-crystal chemical vapor deposition (CVD) graphene. The devices are based on graphene Hall bars with a carrier mobility of >12000 cm^2^ V^–1^ s^–1^ and a low residual carrier density of ∼1 × 10^11^ cm^–2^, showing Hall sensitivity higher than 5000 V A^–1^ T^–1^, which is a value previously only achieved when using exfoliated graphene encapsulated with flakes of hexagonal boron nitride. We also implement a facile and scalable polymeric encapsulation, allowing the performance of graphene Hall bars to be stabilized when measured in an ambient environment. We demonstrate that this capping method can reduce the degradation of electrical transport properties when the graphene devices are kept in air over 10 weeks. State-of-the-art performance of the realized devices, based on scalable synthesis and encapsulation, contributes to the proliferation of graphene-based Hall sensors.

## Introduction

Hall effect sensors are extensively used for proximity sensing, accurate positioning, switching, angular sensing, speed detection, and current sensing, thus being of crucial importance in automotive, aeronautics, consumer electronics, Internet of Things (IoT), and robotic applications.^1−3^ The global Hall sensor market size was estimated to be worth USD ∼1.6 billion in 2022 and to rise at a considerable rate for the next 6 years.^4^ To date, silicon-based Hall sensors are dominating in most applications thanks to well-developed silicon complementary metal-oxide-semiconductor (CMOS) technology and low fabrication costs. However, the room temperature (RT) current sensitivity *S*_*I*_ of silicon Hall sensors is typically limited to ∼100 V A^–1^ T^–1^,^5^ which restricts their applicability. Higher sensitivity can be obtained when using III–V semiconductor materials,^5,6^ which, however, imply higher fabrication costs. Graphene, thanks to its ability to achieve extremely low carrier densities and exceptional mobility,^7,8^ has emerged as an appealing material for the development of highly sensitive cost-effective Hall sensors that are compatible with the existing CMOS platforms. To date, the best-performing graphene Hall sensors have been realized using mechanically exfoliated graphene flakes encapsulated with exfoliated hexagonal boron nitride (hBN), reaching a current-related sensitivity (*S*_*I*_) of ∼5700 V A^–1^ T^–1^.^9^ Hall sensors based on ultraclean exfoliated graphene/hBN stacks were also studied at cryogenic temperatures.^10^ RT measurements (presented in the Supporting Information of ref (10)) indicate high *S*_*I*_ ∼ 8000 V A^–1^ T^–1^ in these devices. Indeed, hBN encapsulation is a key ingredient for the realization of highly performing graphene-based devices because it protects graphene from contaminants and ensures the achievement of record carrier mobility.^11^ Exposure of graphene-based Hall sensors to air leads to decreasing *S*_*I*_ over time due to the physical adsorption of oxygen or water molecules.^12^ Devices based on exfoliated flakes of graphene and hBN, such as the one presented in ref (9), demonstrate high performance, but this approach cannot be considered for industrial production because the size of the exfoliated flakes is typically limited to tens of microns. This issue has been addressed using graphene synthesized via chemical vapor deposition (CVD) on Cu, which has been produced on a wafer scale and successfully integrated into the semiconductor processing lines.^13^ To date, Hall sensors based on CVD graphene have shown *S*_*I*_ ∼ 1200 V A^–1^ T^–1^^14^ and remarkable magnetic resolution.^15^ Scalable encapsulation of graphene, however, remains a challenge to be solved. Graphene Hall sensors with CVD hBN encapsulation have shown lowered sensitivity (i.e., *S*_*I*_ ∼ 97 V A^–1^ T^–1^),^16^ although more promising results (i.e., *S*_*I*_ ∼ 1986 V A^–1^ T^–1^) have been obtained when using CVD hBN as a substrate below the graphene layer.^17^ Although graphene Hall elements (GHEs) display enticing prospects, the demonstration of graphene-based magnetic probes that are both highly sensitive and stable over time, while being scalable, is currently lacking (Table 1).

**Table 1:** Comparison of Figures of Merit for Graphene Hall Sensors Fabricated with Different Approaches and the Data Measured in This Work

type	*S*_*I*_ (V A^–1^ T^–1^)	μ (cm^2^ V^–1^ s^–1^)	n* (cm^–2^)	potential scalability
exfoliated graphene encapsulated with exfoliated hBN^9^	5700	N/A	N/A	no
exfoliated graphene encapsulated with exfoliated hBN^10^	8000	N/A	5 × 10^9^ (*T* = 4.2 K)	no
polycrystalline CVD graphene on SiO_2_^14^	1200	5000	1.1 × 10^12^	yes
polycrystalline CVD graphene encapsulated with CVD hBN^16^	97	1200	N/A	yes
exfoliated graphene encapsulated with exfoliated hBN^39^	5000	>10^5^	6 × 10^10^	no
polycrystalline CVD graphene encapsulated with exfoliated hBN^46^	345	133	N/A	no
single-crystal CVD graphene on SiO_2_^38^	2745	7800	1.75 × 10^11^	yes
single-crystal CVD graphene on SiO_2_ encapsulated with SU-8^47^	800	5100	N/A	yes
single-crystal CVD graphene encapsulated with exfoliated hBN on Kapton^48^	2270	∼17000	N/A	no
this work	∼5030 (∼2130 after 10 weeks)	∼12000	9.5 × 10^10^	yes

**Figure 1:**
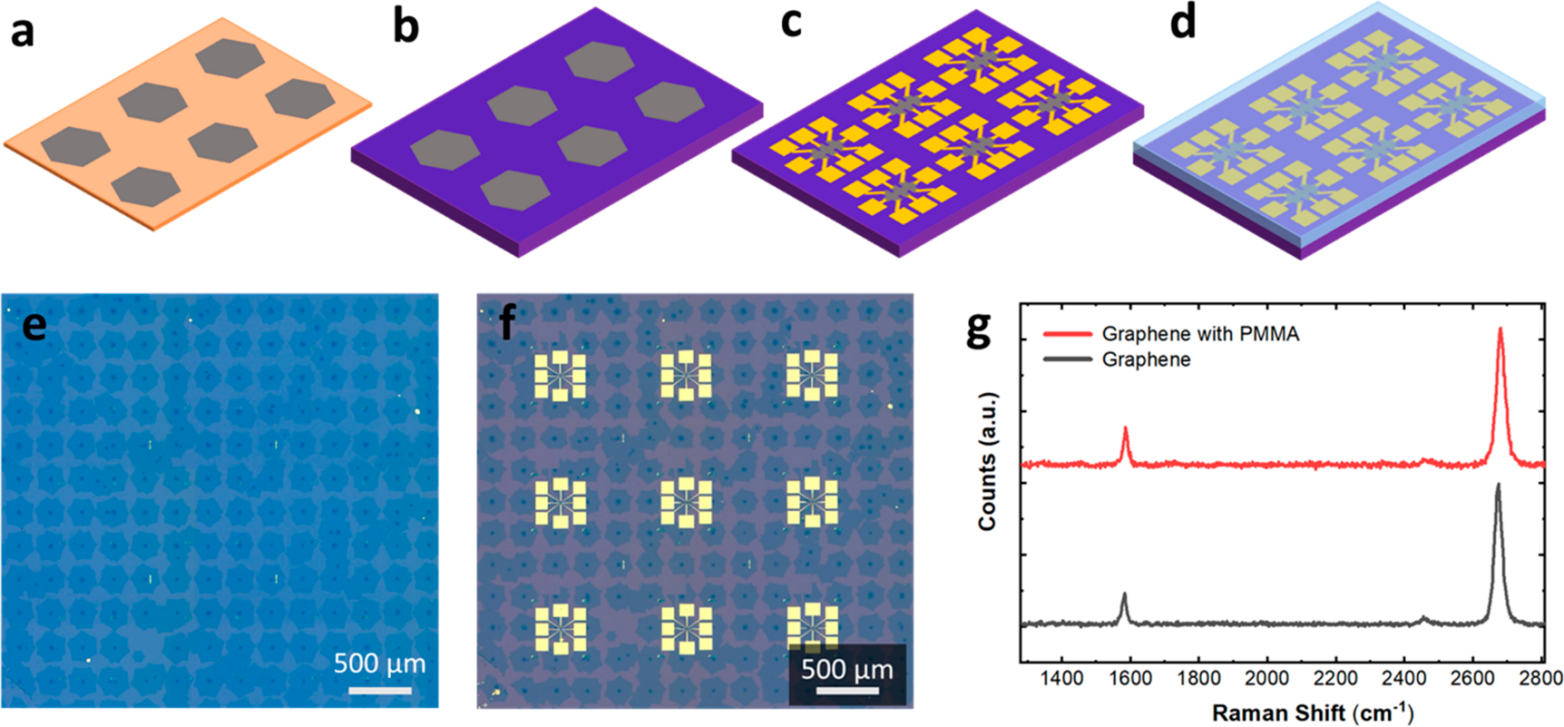
Schematic drawing of a typical array of graphene single crystals (a) grown via CVD on Cu foil, (b) transferred to Si/SiO_2_, (c) with fabricated Hall sensors, and (d) after PMMA spin coating. (e) Optical micrograph of a graphene crystal array transferred on a Si/SiO_2_ substrate. (f) Hall bars fabricated on a graphene crystal array. (g) Representative Raman spectra of bare graphene (bottom) and PMMA-capped graphene (top).

In this work, we demonstrate a facile and systematic approach to achieve high-sensitivity and scalable graphene Hall sensors directly on the technologically relevant substrate Si/SiO_2_, whose performance over time can be stabilized using a polymeric encapsulation. Scalability is ensured by the adoption of graphene single-crystal arrays grown via CVD (Figure 1a), which can be used for wafer-scale integration in several applications.^18,19^ Indeed, CVD-grown graphene single crystals display electronic transport properties comparable to those achieved with mechanically exfoliated graphene flakes,^20^ thus making the realization of scalable ultrahigh-sensitivity GHEs realistic.^20,21^ The fabricated Hall sensors demonstrate carrier mobility exceeding 10^4^ cm^2^ V^–1^ s^–1^ and low charge inhomogeneity at the charge-neutrality point (CNP) n* ∼ 1 × 10^11^ cm^–2^. The devices have high current-related sensitivity with an *S*_*I*_ of up to 5030 V A^–1^ T^–1^. Until now, similar values have been reported only in samples encapsulated with exfoliated hBN, which is not an approach compatible with scalable integration. We also investigate the air stability of graphene Hall sensors without encapsulation over a time of 10 weeks and find a significant degradation in the observed performance. To address this issue, we demonstrate a scalable encapsulation with a polymeric protective layer, poly(methyl methacrylate) (PMMA). PMMA, like hBN,^22^ parylene,^23^ and NaCl,^24^ has been used as an encapsulant of graphene^25,26^ and other 2D materials.^27^ The environmental doping effect on the performance of graphene Hall sensors protected with PMMA is studied by electrical transport measurements with and without the presence of a magnetic field. PMMA-capped graphene Hall sensors display reduced performance degradation such as reduced hysteresis and doping. The scalable and facile approach presented in this work can open a realistic path to the fabrication of highly sensitive and air-stable CVD graphene Hall sensors.

## Results and Discussion

**Figure 2:**
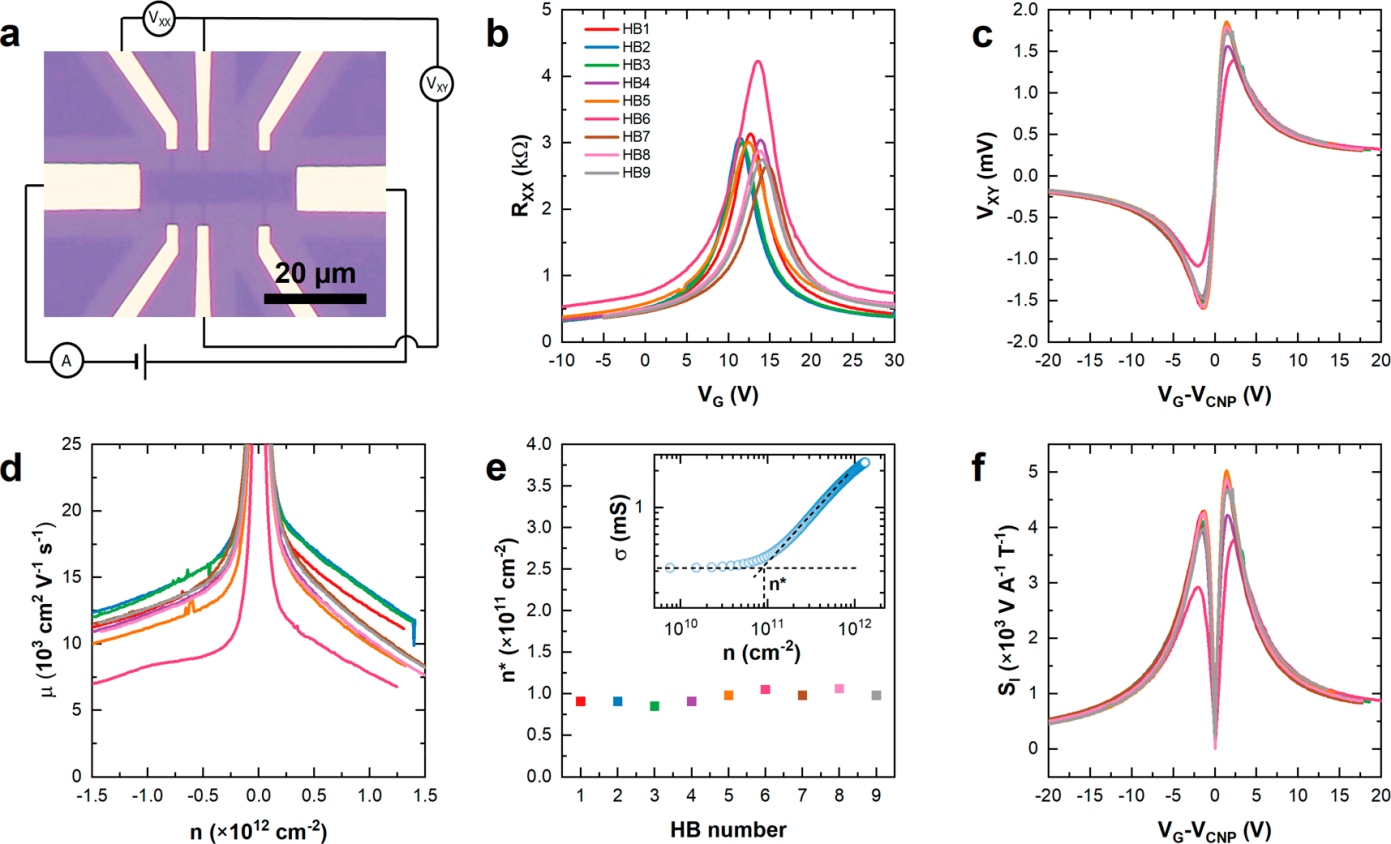
Electrical transport properties of chip 1. (a) Electrical diagram of a typical transport measurement. (b) Resistivity as a function of the applied gate voltage. Different colors represent nine different Hall bars. (c) Hall voltage as a function of the applied gate voltage with respect to the CNP. (d) Carrier mobility as a function of the carrier density. (e) Charge inhomogeneity at the CNP. Inset: Conductivity of a representative device (HB2) as a function of the carrier density on a double-logarithmic scale. A linear fit is used to obtain n*. (f) Current-related sensitivity as a function of the applied gate voltage obtained at *B* ∼ 0.37 T.

Arrays of hexagonal graphene crystals (sketched in Figure 1a and shown in Figure 1e) were grown via CVD on Cu foil using Cr nucleation seeds^19^ and subsequently transferred from the growth substrate to SiO_2_/Si using a semidry transfer technique^18^ (Figure 1b). The lateral size of the crystals was ∼150 μm with a predominantly monolayer thickness (confirmed using Raman spectroscopy; Figure 1g). We used deterministic transfer to accurately align the graphene crystal array on the target substrate, ensuring that our device channels avoided small multilayer areas present near the nucleation points of the crystals^19,28^ and potential grain boundaries caused by the merging of adjacent crystals. Device fabrication was performed using electron beam lithography (EBL), reactive ion etching (RIE), and thermal evaporation of metals (Figure 1c,f; see Methods for further details). Where applicable, the devices were finally capped with a PMMA layer, as shown in Figure 1d. Figure 2a shows an optical micrograph of a typical device and an electrical diagram of a transport measurement. The device has a Hall bar geometry, with a total length of ∼30 μm and a width of ∼6 μm. During four-terminal transport measurements, adjacent lateral contacts with 6 μm spacing were used to probe the voltage drop. In some cases, the resistivity measurements were cross-checked by using a different pair of lateral contacts with a 12 μm spacing. The width of the lateral (probe) contacts was optimized for high sensitivity and was set to ∼1 μm to keep the Hall channel smaller than the main channel and minimize the Hall voltage offset caused by inhomogeneities.^1,29^

Raman spectroscopy was performed on the samples to assess the layer number, crystallinity, and doping of graphene before and after PMMA deposition. Raman maps were obtained on the graphene crystals near the Hall bars. Figure 1f shows representative Raman spectra obtained before (black curve) and after (red curve) PMMA deposition. The spectra show two prominent Raman peaks (G and 2D), which can be fit with a single Lorentzian function, corresponding to single-layer graphene. Before PMMA deposition, the average peak position is found at 1581.5 ± 0.9 cm^–1^ (G peak) and ∼2673 ± 1.8 cm^–1^ (2D peak). The full-width-at-half-maximum (fwhm) values are fwhm(G) ∼ 15.3 ± 1.2 cm^–1^ and fwhm(2D) ∼ 25.5 ± 1.5 cm^–1^. The D peak, typically observed near 1350 cm^–1^ in defective graphene,^30^ is absent, indicating a high crystalline quality of our material. The 2D/G peak intensity ratio *I*(2D)/*I*(G) is ∼4.7 ± 0.6 and the area ratio *A*(2D)/*A*(G) is ∼7.8 ± 1, both indicating doping of ∼10^12^ cm^–2^.^31^ The Raman fitting values obtained in this sample indicate high-quality graphene, which is a result of a monocrystalline material,^19^ semidry transfer,^18^ and two-step cleaning^32^ adopted during the device fabrication process. After PMMA deposition, the average positions of both peaks are blue-shifted to Pos(G) ∼ 1583.9 ± 1 cm^–1^ and Pos(2D) ∼ 2679.0 ± 2 cm^–1^. The other fitting parameters show a small change (less than the standard deviation): fwhm(G) ∼ 15.0 ± 1.3 cm^–1^, fwhm(2D) ∼ 25.6 ± 1.2 cm^–1^, *I*(2D)/*I*(G) ∼ 4.8 ± 0.6, and *A*(2D)/*A*(G) ∼ 8.2 ± 1. This indicates that polymer deposition has a very small effect on doping or submicron-scale strain variation of the sample, and we can attribute the change of peak positions^33,34^ to a small increase of overall uniaxial (biaxial) strain by ∼0.1% (0.05%). Full Raman peak correlation data are presented in Figure S2.

Our graphene arrays have previously been used for the wafer-scale fabrication of photonic devices (up to 150 mm)^18^ and Hall bars.^32^ The set of nine Hall bars (HB1–HB9) fabricated on chip 1 and shown in Figure 1f were fully characterized electrically. Electrical transport characteristics of the nine Hall bars, plotted in Figure 2, were determined by performing field-effect measurements in ambient conditions. As shown schematically in Figure 2a, source–drain current *I*_SD_ = 1 μA was applied along the channel, while sweeping the back gate voltage *V*_G_ and measuring the longitudinal voltage *V*_*XX*_. Sample resistivity *R*_*XX*_, shown in Figure 2b, was determined using the relationship *R*_*XX*_ = *V*_*XX*_/*I*_SD_. As-fabricated, the charge neutrality point *V*_CNP_ of the devices were found to be near gate voltage *V*_G_ ∼ 13 V, corresponding to low p-type doping of ∼1 × 10^12^ cm^–2^. The response of our devices to a magnetic field was measured to investigate their potential use as Hall sensors. Using a permanent magnet, a fixed perpendicular magnetic field *B* ∼ 0.37 T was applied to the Hall bars, while passing constant current of 1 μA. The Hall voltage *V*_*XY*_ as a function of the applied back gate voltage was measured and is shown in Figure 2c. The carrier mobility μ, shown in Figure 2d, was determined using the Drude formula μ = 1/ρ*ne*, where ρ is the sample resistivity, equal to *R*_*XX*_ due to the square geometry of the channel, *n* is the gate-induced carrier density, determined from gate capacitance,^12,35^ and *e* = 1.6 × 10^–19^ C is the elementary charge. μ values were estimated at a technologically relevant carrier density *n* = 1 × 10^12^ cm^–2^. The average μ was found to be ∼(1.2 ± 0.1) × 10^4^ cm^2^ V^–1^ s^–1^, with HB2 showing μ as high as ∼1.4 × 10^4^ cm^2^ V^–1^ s^–1^. Figure 2e shows the residual carrier density at the CNP, which was determined from a linear fit of the conductivity as a function of the carrier density on a logarithmic scale.^36^ The inset of Figure 2e shows an example of the fit for HB2. Full n* fitting data is shown in Figure S3. The average value for the nine devices was found to be n* ∼ (9.5 ± 0.5) × 10^10^ cm^–2^. We estimate the current-related sensitivity *S*_*I*_ of our graphene Hall sensors, which is calculated using the following equation: *S*_*I*_ = *V*_*XY*_/*IB*, at a constant current *I* = 1 μA and magnetic field *B* = 0.37 T perpendicular to the channel.^1^ The average *S*_*I*_ was estimated to be *S*_*I*_ ∼ 4642 ± 392 V A^–1^ T^–1^, with HB5 showing *S*_*I*_ ∼ 5030 V A^–1^ T^–1^. The high *V*_*XY*_ and *S*_*I*_ values measured in our devices are obtained because of the high μ and the remarkably low n* (for CVD graphene on SiO_2_), which allows us to reach a low single-type carrier density in the vicinity of the CNP, a well-known prerequisite for the high Hall coefficient.^37−39^ Indeed, as can be seen in Figure S3, we observed slight differences in the n* values estimated on the hole and electron branches of the field-effect curves of some devices, which agree well with the small V_*XY*_ asymmetry measured in the corresponding Hall bars. To the best of our knowledge, this array of devices demonstrates the highest *S*_*I*_ measured in Hall sensors based on CVD graphene^14,40^ and is approaching the sensitivity observed in Hall sensors based on exfoliated graphene with hBN encapsulation.^9,39^

As-fabricated devices show a small spread in resistivity (Figure 2b), Hall voltage (Figure 2c), and current-related sensitivity (Figure 2f). It is well-known that the electrical performance of bare graphene-based devices measured in ambient conditions can be strongly affected by atmospheric adsorbents and that graphene encapsulation is beneficial for achieving a homogeneous performance.^41^

**Figure 3:**
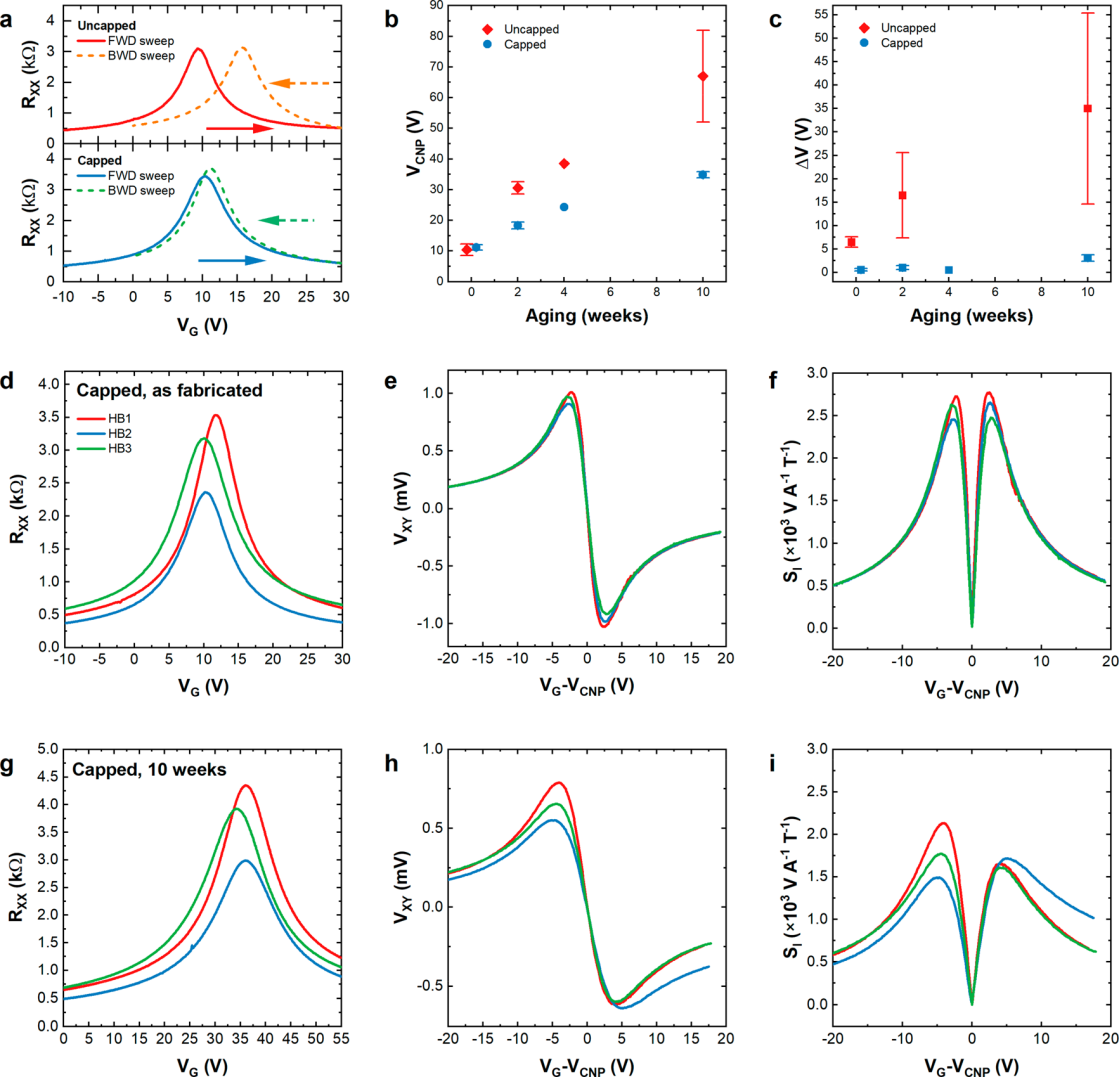
Electrical characterization of graphene Hall bars without and with PMMA encapsulation. (a) Hysteresis of resistivity measurements as a function of the applied gate voltage. (Top) Uncapped Hall bar: forward (red solid) and backward (orange dashed) back gate sweeps. (Bottom) PMMA-capped Hall bar: forward (blue solid) and backward (green dashed) back gate sweeps. (b) Position of the CNP and (c) hysteresis of the uncapped (red) and capped (blue) graphene devices as a function of the aging time. (d–f) Electrical performance of chip 3 after PMMA encapsulation, showing resistance (d), Hall voltage (e), and current-related sensitivity (f) as a function of the applied gate voltage. (g–i) Electrical performance of chip 3 after 10 weeks of aging, showing resistance (g), Hall voltage (h), and current-related sensitivity (i) as a function of the applied gate voltage.

To further study the device stability in ambient conditions and the effect of air contaminants on the Hall bar performance, we have fabricated two other chips (chips 2 and 3) with arrays of graphene Hall bars. Both chips were characterized initially to verify their quality, obtaining an average μ of ∼10^4^ cm^2^ V^–1^ s^–1^ in each case. Chip 3 was then capped with a thin (∼240 nm) layer of PMMA via spin coating, as described in Methods. In Figure 3a, we compare the hysteresis of field-effect measurements on uncapped and capped graphene. The top panel shows the field effect measured in chip 2 (uncapped). Sweeping the back gate voltage from −10 to +30 V (red solid curve), we measure *V*_CNP_ ∼ 9.5 V, while on the back sweep (orange dashed curve), *V*_CNP_ is shifted to ∼15.8 V, with a hysteresis Δ*V* ∼ 6.3 V. In the bottom panel, we show the measurements obtained on chip 3 (capped). On the forward sweep (−10 to +30 V, blue solid curve), we measure *V*_CNP_ ∼ 10 V, while sweeping back to 0 V (green dashed curve), we measure *V*_CNP_ ∼ 10.9 V, which indicates a strongly reduced effect of atmospheric adsorbents on the electrical characteristics of this device. Chips 2 and 3 were then stored under ambient conditions (temperature ∼25 °C; relative humidity ∼50%) for 10 weeks. To study the trend of aging, we have performed field-effect measurements at two intermediate time periods, i.e., after 2 and 4 weeks. Figure 3b shows the average *V*_CNP_ obtained for chip 2 (uncapped, red) and chip 3 (capped, blue) at various stages of aging. For the uncapped chip, there is a strong shift of *V*_CNP_ over time, reaching the average *V*_CNP_ ∼ 30.6 ± 2 V after 2 weeks, 38.5 V after 4 weeks, and ∼65 ± 15 V after 10 weeks. There is also pronounced hysteresis in this chip (shown in Figure 3c, red): Δ*V* ∼ 16.5 ± 9 V after 2 weeks and Δ*V* ∼ 35 ± 20.5 V after 10 weeks. The strong increase in p-type doping, high hysteresis, and pronounced device-to-device variation indicate that uncapped graphene is strongly susceptible to atmospheric adsorbents, making repeated measurements hardly reproducible. This is also observed in Figure S4a, where a continuous shift of *V*_CNP_ is seen over repeated back gate sweeps. In contrast, in the PMMA-capped sample, repeated gate sweeping has little effect on the *V*_CNP_ position (Figure S4b). The role of PMMA capping in chip 3 is further demonstrated when studying the effect of aging (Figure 3b). The position of *V*_CNP_ is increased over time (*V*_CNP_ ∼ 18.3 ± 1.1 V after 2 weeks, ∼24.3 V after 4 weeks, and ∼35 ± 1 V after 10 weeks), although the shift is much smaller than that for chip 2. Even more strikingly, as can be seen in Figure 3c, chip 3 shows almost negligible hysteresis over the whole aging period. Δ*V* remains ∼1.0 V even after 4 weeks, finally reaching a maximum average Δ*V* ∼ 3.0 ± 0.7 V after 10 weeks. This is particularly important because small hysteresis is crucial to obtain the reproducible performance of sensors. Figure S5 shows the effect of PMMA encapsulation on the transport properties of a representative device on chip 3 and full evolution of field-effect characteristics over the 10-week investigation period.

Parts d–i of Figure 3 also show the electrical performances of three representative Hall bars (HB1–HB3) on chip 3. We plot the resistivity (Figure 3d), Hall voltage (Figure 3e), and *S*_*I*_ (Figure 3f) after device fabrication and PMMA capping. Compared to chip 1 (Figure 2), the electrical performance of the sample is slightly reduced, but device-to-device variation is small. Even after 10 weeks, we measure the resistivity (Figure 3g), Hall voltage (Figure 3h), and *S*_*I*_ (Figure 3i) values, which compare well with the state-of-the-art performance of devices based on CVD graphene. Table 1 compares our results to other Hall sensors presented in the literature, listing Figures of Merit such as *S*_*I*_, μ, and n* (where available), as well as the compatibility of the adopted materials with wafer-scale fabrication.

It should be mentioned that PMMA encapsulation is reported in this work to exemplify the positive effect that a simple encapsulant not requiring highly specialized equipment (i.e., only a simple table-top spin coater and a hot plate) can have on the performance of Hall sensors. The reported approach provides a simple way to stabilize the fabricated devices for ambient environment measurements, with a possibility for reversible capping (i.e., it can be completely removed from graphene using two-step cleaning).^32^ Other polymers with a lower water vapor transmission rate could provide an even more effective environmental barrier. Graphene encapsulated with Parylene C, in combination with a metallic overlayer, has shown excellent stability in air over several months,^42^ and such an approach could be suitable for protecting graphene Hall sensors. In the field of graphene, oxide dielectrics such as Al_2_O_3_ or HfO_2_ are often used as encapsulants,^43^ although their deposition needs specialized equipment and the necessary process conditions (such as relatively high temperature or plasma) may affect the electrical transport properties of graphene. For high-quality graphene encapsulation, hBN remains the optimum material, providing not only environmental protection but also an ultraflat surface with a homogeneous potential. Exfoliated hBN lacks scalability, but recent progress in the synthesis of wafer-scale hBN has shown promising electrical transport results^44^ and could become a viable encapsulant for high-performing scalable graphene Hall sensors. Furthermore, the integration of Hall sensors in electronics typically requires the semiconductor chips to be embedded in packaging. Industrially produced graphene Hall sensors could adopt hermetic packaging^45^ in a vacuum or an inert atmosphere to mitigate the aging issues.

## Conclusion

In conclusion, we have demonstrated a facile and scalable approach to fabricating highly sensitive graphene-based Hall sensors. As-fabricated, our devices have a high average carrier mobility of ∼1.2 × 10^4^ cm^2^ V^–1^ s^–1^ with a low residual charge carrier density of <1.0 × 10^11^ cm^–2^. This allows us to reach a high Hall voltage, corresponding to an average current-related Hall sensitivity of ∼4600 V A^–1^ T^–1^ (with the best-performing device showing >5000 V A^–1^ T^–1^), which is a record value for devices based on CVD graphene. We use a polymeric (PMMA) capping to minimize the effects of atmospheric adsorbents, thus ensuring a stable performance (i.e., low hysteresis of <1 V) of our devices even when measured in ambient conditions. Furthermore, PMMA capping slows down the degradation of the device performance even when stored in air for prolonged times (up to 10 weeks). Our work demonstrates that monocrystalline CVD graphene on Si/SiO_2_ can be a viable material for the scalable production of Hall sensors whose performance exceeds those fabricated using conventional technology.

## Methods

Graphene arrays were synthesized on Cu foil via CVD using deterministic seeding,^19^ which yields single crystals around each nucleation point, as confirmed by selected-area electron diffraction measurements.^19^ Briefly, optical lithography and thermal evaporation were used to deposit an array of Cr nucleation seeds matching the geometry of the Hall sensor array on electropolished Cu foil (Alfa Aesar 46365, purity 99.8%). CVD growth was performed in a cold-wall CVD reactor (Aixtron BM Pro) at a temperature of 1060 °C under a flow of Ar (900 sccm), H (100 sccm), and methane (1 sccm). A sample enclosure was utilized to control the nucleation density.^49^ The array of graphene single crystals was electrochemically delaminated from Cu foil, aligned to the markers present on the target substrate, and deposited in dry conditions, as described previously.^18^ The Hall bars were fabricated by using EBL and RIE at 35 W with a flow of 5 sccm of Ar and 80 sccm of O_2_. Subsequently, electrical contacts were designed via EBL and deposited by thermal evaporation of 50 nm Au with a 5 nm Cr adhesion layer. PMMA (AR-P 672.045, Allresist GMBH) was used for lithography and encapsulation. It was spin-coated on graphene with 4000 rpm for 60 s. For lithography steps, PMMA was baked at 120 °C for 5 min on a hot plate, whereas for encapsulation, the baking temperature and time were increased to 160 °C and 15 min, respectively, to ensure complete evaporation of the solvent. The PMMA layer thickness was measured by using a Bruker stylus profilometer. After transfer and each fabrication step, we adopted a two-step cleaning process demonstrated previously.^32^ Namely, bulk polymers were removed in acetone for at least 2 h, followed by rinsing in isopropyl alcohol. Remover AR 600-71 (Allresist GMBH) was then used for 3 min to remove the nanoscale polymeric residues. Finally, the samples were rinsed with deionized water. After device fabrication, Raman spectroscopy was performed with a Renishaw InVia system with a 532 nm laser and 100× objective. The laser power was set to ∼1 mW to avoid excess heating. Electrical transport measurements were carried out in ambient conditions using microprobes connected to a pair of Keithley 2450 source-measure units in a four-terminal configuration. Hall measurements were carried out by using a permanent U-shaped magnet of 0.37 T (Ecopia MS37R).

## Supporting Information Available

High-magnification optical and atomic force microscopy images of device channels, Raman peak dispersion data, fitting of the charge inhomogeneity for all devices, and additional data regarding sample stability (PDF)
