# Supplementary material for: Highly Sensitive Hall Sensors Based on Chemical Vapor Deposition Graphene
Source: ACS Appl Nano Mater. 2023 Nov 21;7(16):18329–36. doi: 10.1021/acsanm.3c03920 (PMC11348313; doi:10.1021/acsanm.3c03920)
Supplement: Supplementary file 1 — an3c03920_si_001.pdf [file an3c03920_si_001.pdf]

Supporting Information

Highly Sensitive Hall Sensors Based on CVD graphene

Ayush Tyagi[ab*], Leonardo Martini[bc], Zewdu M. Gebeyehu[bc], Vaidotas Mišeikis[bc*], Camilla Coletti[bc*]

[a] NEST, Scuola Normale Superiore, Piazza San Silvestro 12, 56127 Pisa, Italy

[b] Center for Nanotechnology Innovation @NEST, Instituto Italiano di Technologia, Piazza San Silvestro 12, 56127 Pisa, Italy

[c] Graphene Labs, Istituto Italiano di Tecnologia, via Morego 30, 16163 Genova, Italy

* Corresponding authors: camilla.coletti@iit.it, vaidotas.miseikis@iit.it, ayush.tyagi@sns.it

Graphene crystal alignment

We have used deterministic semi-dry transfer to deposit our graphene crystals on the target substrates (n-doped Si with 285 nm of thermal SiO_2) containing prefabricated local alignment markers. To avoid multilayer graphene areas present at the center of some of the graphene crystals, the hexagonal crystals were slightly shifted with respect to the markers, as can be seen in Fig. S1a. Fig. S1b shows a close-up view of an etched graphene Hall bar. The multilayer graphene is evident to the right side of the active device channel. As can be seen in the AFM image in Fig. S1c, the fabricated active channels of the devices are clean and flat. We measured overall average roughness ~0.6 nm on the device channels, with roughness ~0.5 nm in the areas without any wrinkles, which matches the SiO_2 surface roughness declared by the wafer supplier. This indicates a conformal contact of graphene on the substrate and contamination-free interface.

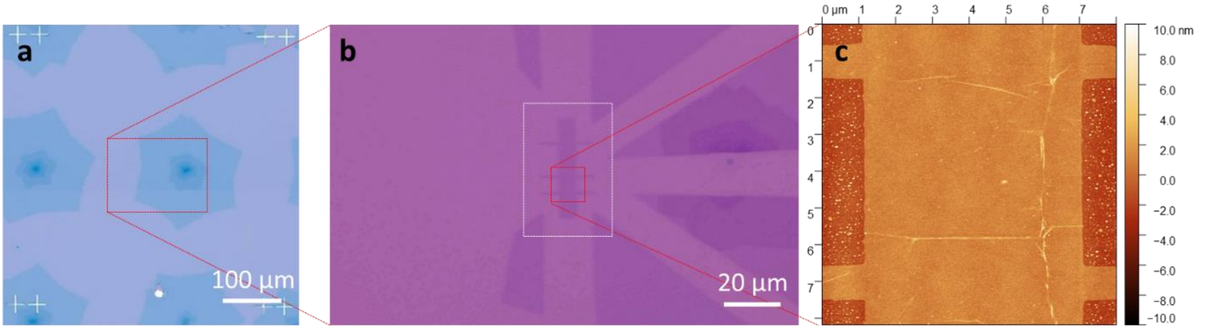

Figure S1. Optical and AFM characterization of graphene during the device fabrication. a) Optical micrograph showing shifted placement of graphene crystal array with respect to the final device positions to avoid multilayer areas. b) Graphene Hall bar after etching. Multilayer area can be seen on the right, away from the device channel. c) AFM image of the Hall bar, showing the clean and flat device surface.

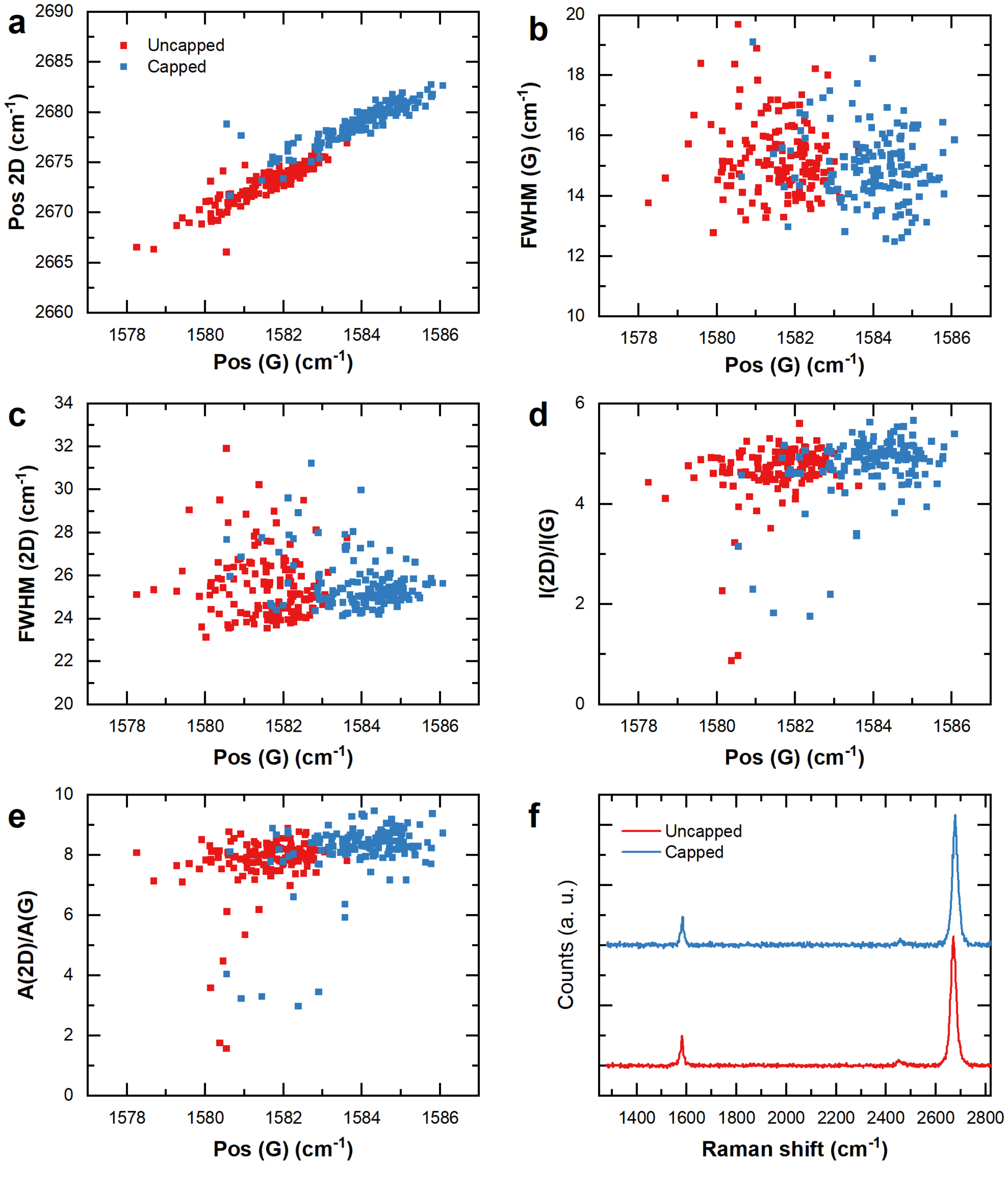

Figure S2. Raman correlation plots for uncapped and PMMA-capped graphene. (a-e) Raman G peak and 2D peak scattering as a function of G peak position for uncapped graphene (red data points) and capped graphene (blue data points). (a) 2D peak position, (b) G peak width, (c) 2D peak width, (d) Intensity ratio of 2D peak and G peak, (e) Area ratio of 2D peak and G peak. f) representative Raman spectra for uncapped graphene (red) and capped graphene (blue).

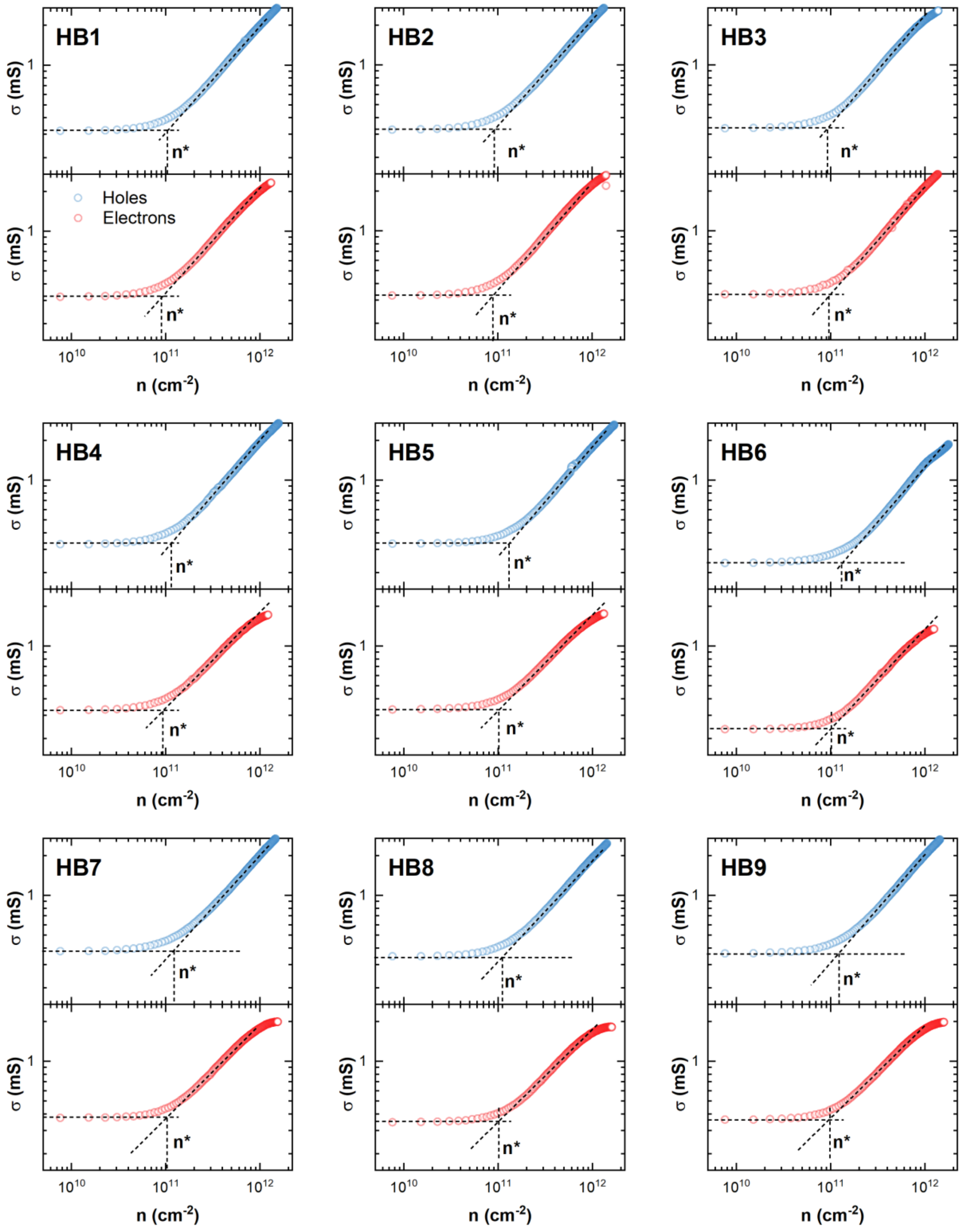

Figure S3. n* estimation for the 9 Hall bars of Chip 1 *via* linear fit of conductivity as a function of carrier density on a double logarithmic scale.

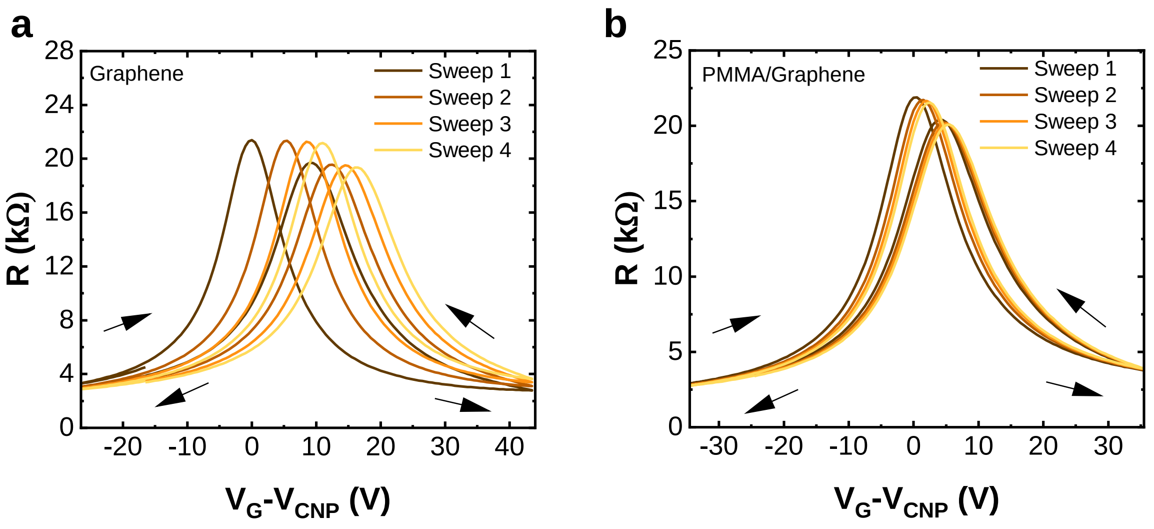

Figure S4. Evolution of transfer characteristics as a function of applied back gate voltage over several back-gate sweeps for the a) uncapped graphene device and b) PMMA-capped device.

To study the stability of graphene Hall sensors further, we have performed repeated sweeping of the back gate while measuring the resistance of the device. As can be seen in Figure S4a, the CNP of the Hall bar with bare graphene keeps shifting towards higher values with each sweep of the applied back gate due to increasing adsorption of O_2 and H_2O molecules on the surface of graphene. The forward sweep CNP is shifted by 11.4 V after 4 sweeps. In contrast, the PMMA-capped device (Figure S4b) shows much more stable transfer characteristics after repeated sweeping of the back gate, with a shift of just 2 V after 4 sweeps. This proves that PMMA is a suitable technique for stable operation of graphene devices in ambient.

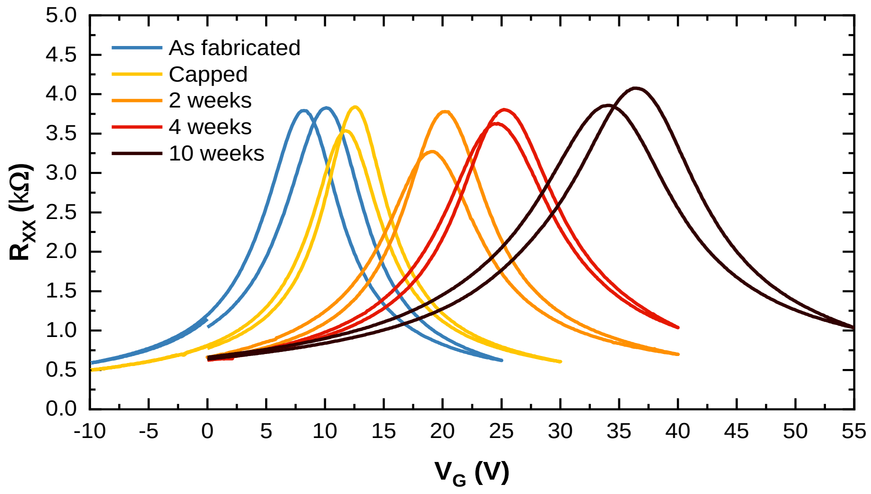

Figure S5. Sample resistivity as a function of applied gate voltage after fabrication (blue curve), after PMMA capping (yellow curve) and its evolution over time (orange-red-black curves).

Atomic force microscopy (AFM) is used to evaluate the PMMA roughness after spin-coating and annealing at 160 °C on top of monolayer graphene/SiO_2. A representative AFM image is shown in Figure S4 (a). Figure S4 (b) depicts the height distribution of 10 × 10 μm^2 of spin-coated PMMA, indicating an RMS roughness ~0.49 nm.

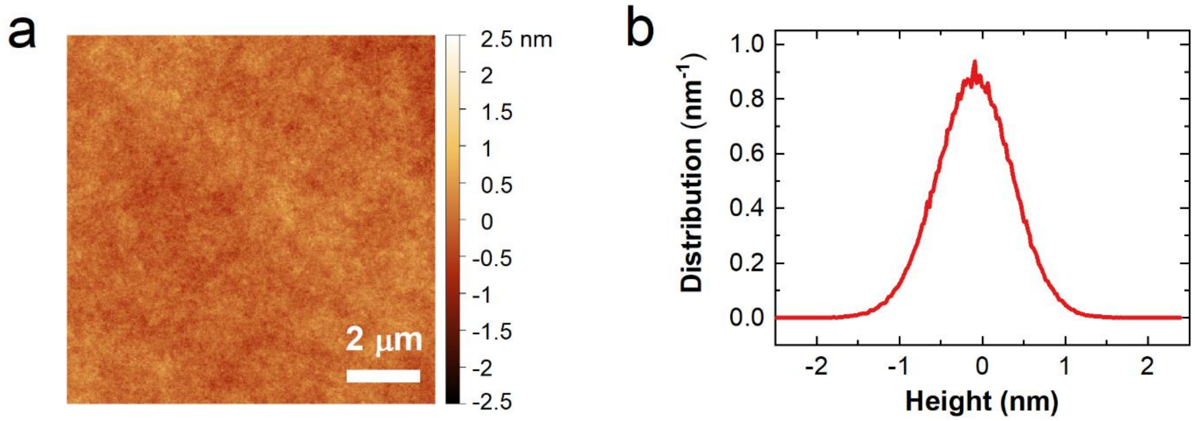

Figure S6. (a) Surface topography AFM image of a 10x10 μm^2 area of PMMA spin-coated on graphene. (b) Height distribution of the sample area.

Recovery of aged device performance in vacuum

We have studied the effect of vacuum storage on the electrical properties of a graphene Hall bar affected by aging. A device was initially measured to have V_{CNP} ~13 V, μ ~11600 cm^2 V^{-1} s^{-1} and S_I ~3750 V A^{-1} T^{-1}. After storing the sample in ambient conditions, the V_{CNP} was shifted to ~33 V, μ decreased to ~5400 cm^2 V^{-1} s^{-1} and SI to ~2540 V A^{-1} T^{-1}. The sample was then placed in a desiccator at rough vacuum ~8 mbar for 7 days and the characterization was repeated. The V_{CNP} was ~23 V, partially recovering towards the initial value, while mobility increased to ~6100 cm^2 V^{-1} s^{-1}. Magnetic sensitivity also increased, reaching ~3270 V A^{-1} T^{-1}. This demonstrates that a sample affected by environmental aging can be partially recovered by storing it in vacuum, though the positive effects of such vacuum storage are weaker than the negative effects of environmental aging, and full performance recovery may not be possible or may need excessively long vacuum storage times. Notably, we did not observe a significant recovery of electrical properties when storing a capped sample in vacuum after aging, though it can reasonably be expected that high or ultra-high vacuum treatment could be more effective at promoting the desorption of atmospheric species even in the presence of capping.

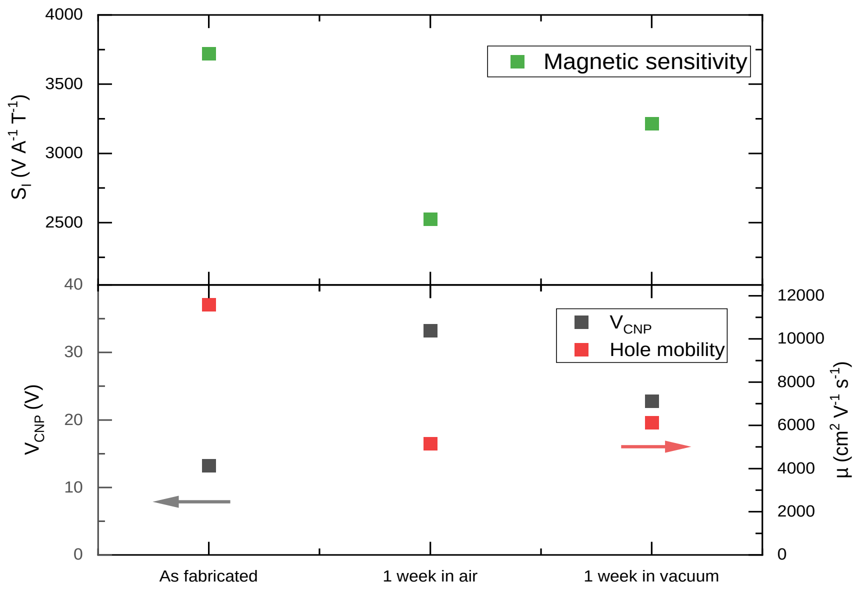

Figure S7. Degradation of device performance in air and subsequent recovery by vacuum storage.
